# Therapeutic Targeting of Th17/Tc17 Cells Leads to Clinical Improvement of Lichen Planus

**DOI:** 10.3389/fimmu.2019.01808

**Published:** 2019-07-31

**Authors:** Farzan Solimani, Robert Pollmann, Thomas Schmidt, Ansgar Schmidt, Xiang Zheng, Rajkumar Savai, Stefan Mühlenbein, Julia Pickert, Verena Eubel, Christian Möbs, Rüdiger Eming, Michael Hertl

**Affiliations:** ^1^Department of Dermatology and Allergology, Philipps-Universität Marburg, Marburg, Germany; ^2^Department of Pathology, Philipps-Universität Marburg, Marburg, Germany; ^3^Max Planck Institute for Heart and Lung Research, Member of the German Center for Lung Research (DZL), Member of the Cardio-Pulmonary Institute (CPI), Bad Nauheim, Germany; ^4^Department of Internal Medicine, Member of the DZL, Member of CPI, Justus Liebig University, Giessen, Germany

**Keywords:** lichen planus, IL-17, secukinumab, ustekinumab, guselkumab, T cells

## Abstract

Lichen planus (LP) is a common, chronic relapsing inflammatory disorder of the skin and mucous membranes which often poses a major therapeutic challenge due to its refractory course. Novel pathogenesis-based therapies are urgently needed. As several studies have shown that IL-17 may contribute to LP pathogenesis, we investigated whether therapeutic targeting of IL-17^+^ T cells leads to clinical improvement of mucosal and cutaneous LP lesions. A total of five patients with lichen planus were treated in a compassionate use trial with either secukinumab (anti-IL-17; 3 patients with acute and chronic recalcitrant muco-cutaneous LP), ustekinumab (anti-IL-12/IL-23; 1 patient with recalcitrant oral LP) or guselkumab (anti-IL-23; 1 patient with recalcitrant oral LP). The clinical course of the patients was assessed by the Autoimmune Bullous Skin Disorder Intensity Score (ABSIS) reflecting both extent and severity of disease and functional sequelae of oral involvement for at least 12 weeks. The inflammatory infiltrate in lesional and post-lesional skin was analyzed by immunohistochemistry before and after treatment. Furthermore, the cytokine profile of peripheral blood T cells from the treated patients was assessed by flow cytometry and/or ELISpot assay. Treatment with secukinumab induced rapid and prolonged clinical amelioration of muco-cutaneous LP. Clinical improvement was accompanied by a strong reduction of the Th1 and Th17/Tc17 cellular mucosal and cutaneous infiltrate. Moreover, long-term treatment of one patient with recalcitrant oral LP with ustekinumab led to healing of the ulcerative oral lesions and a reduction of peripheral blood and lesional IL-17^+^ T cells. Finally, treatment with guselkumab led to a marked clinical improvement in a patient with recalcitrant erosive oral LP. These findings show for the first time that therapeutic targeting of Th17/Tc17 cells leads to a pronounced clinical amelioration of mucosal and cutaneous LP and strongly suggests that IL-17-producing T cells are central to disease pathogenesis. Thus, therapeutic targeting of Th17/Tc17 cells opens new therapeutic avenues in the treatment of recalcitrant LP.

## Introduction

Lichen planus (LP) is a common chronic relapsing inflammatory skin disease of the mucous membranes and the skin which presents with pruritic papules and painful plaques on the skin and erosion and ulcers on the mucous membranes (1–3) while in lichen planopilaris, a follicular form of LP, the scalp can also be affected (4). Although common, the therapeutic options, particularly in mucosal LP, are rather limited due to a mostly chronic refractory course (3). Systemic immunosuppressants, such as glucocorticoids, ciclosporin, azathioprine, and methotrexate, or immunomodulators such as acitretin, help to ameliorate clinical symptoms but have considerable side effects upon long-term treatment. The inflammatory skin infiltrate of LP is characterized by a dense dermal T cell-dominated cellular infiltrate (1). At present, the target antigens of the T cellular response in LP are poorly characterized. Human papilloma virus (HPV) and hepatitis C virus have been implicated as etiologic factors in selected cases of oral LP (1). Our group has recently identified autoreactive Th1 and Th17 cell responses against bullous pemphigoid (BP) antigen 180, a well-known autoantigen of the skin, in LP patients with mucocutaneous involvement. Of note, IL-17-producing cells were present within the inflammatory skin infiltrate underneath the dermal epidermal basement membrane zone (BMZ) where apoptotic epidermal keratinocytes are typically seen in LP (5). In addition, different groups demonstrated the presence of Th17 cells and Th17-related cytokines in LP lesions (6–9). These findings strongly suggest a potential role of IL-17 cells in the LP pathogenesis and raise the question as to whether therapeutic targeting of IL-17 or IL-17-producing T cells leads to an amelioration of LP.

## Methods

### Patients

Three patients with mucocutaneous LP were treated with the anti-IL-17A monoclonal antibody, secukinumab, one LP patient with recalcitrant oral LP was treated with the anti-p40 monoclonal antibody, ustekinumab, which targets the p40 subunit of both IL-23 and IL-12 and one LP patient with chronic lesions not responding to immunosuppressive treatments was treated with guselkumab, a monoclonal antibody targeting IL-23. The patients' characteristics are shown in Table 1. Diagnosis of LP was based on the clinical phenotype and histopathological findings. None of the studied LP patients were on systemic immunosuppressive treatment including glucocorticoids. All patients had an extensive clinical manifestation of LP with either severe widespread skin involvement (Patient 1) or chronic recalcitrant oral involvement (Patients 2–5) which did not sufficiently respond to standard medical care, i.e., topical and systemic glucocorticoids (Table 1).

**Table 1:** Synopsis of studied lichen planus patients.

**P**	**Sex (m/f)**	**Age (y)**	**Clinical phenotype**	**Disease duration**	**Study drug**	**Treatment and observation period**	**Previous treatment**
1	f	25	Disseminated cutaneous polygonal papules on the trunk and extremities, extensive erosions of the oral, and vaginal mucosa	3 months	Secukinumab 300 mg s.c. at weeks 0,1,2,3,4 followed by monthly treatment	12 weeks	none
2	f	56	Palmoplantar confluent polygonal papules and plaques, moderate erosions of the oral mucosa	6 months	Secukinumab 300 mg s.c. at weeks 0,1,2,3,4 followed by monthly treatment	12 weeks	Topical corticosteroids
3	f	58	Extensive erosions of the gingivae, tongue, buccal mucosa, and the palatine	7 years	Secukinumab 300 mg s.c. at weeks 0,1,2,3,4 followed by monthly treatment	48 weeks (ongoing)	Ciclosporin, systemic and topical corticosteroids, acitretin, intravenous immunoglobulins
4	f	72	Extensive erosions of the oral mucosa, pronounced painful, and recalcitrant ulcerations of the tongue and gingivae	6 years	Ustekinumab 45 mg s.c. at weeks 0 and 4 and every 3 months thereafter	48 weeks (ongoing)	Ciclosporin, systemic and topical corticosteroids, acitretin, azathioprine
5	f	72	Recalcitrant ulcerations of the tongue	5 years	Guselkumab 100 mg s.c. at weeks 0 and 4 and every 2 months thereafter	30 weeks (ongoing)	Systemic and topical corticosteroids

Secukinumab, ustekinumab, and guselkumab are approved for the treatment of psoriasis and were administered according to the guidelines for psoriasis treatment (10–12) for at least 12 weeks (Table 1). LP skin lesions were initially treated with a short course of topical glucocorticoids for up to 7 days followed by plain emollients. Oral LP lesions were only treated with rinsing solutions containing antiseptics and local anesthetics. Off-label treatment with secukinumab, ustekinumab, and guselkumab, respectively, was covered by a compassionate use program aimed at identifying novel effective treatment options in refractory LP and did not require formal approval by the local ethics committee. The clinical course of the patients was assessed by the Autoimmune Bullous Skin Disorder Intensity Score (ABSIS) (13) which reflects both extent and severity of disease and functional sequelae of oral involvement.

Punch biopsies from lesional and post-lesional skin were obtained from patient 1–4 before and at week 12 of treatment which were fixed in paraformaldehyde, embedded in paraffin, and further processed for immunohistochemical analysis of the inflammatory infiltrate as recently described (5). Peripheral blood mononuclear cells of the patients were isolated from citrate-phosphate-dextrose-adenine (CPDA)-treated blood samples and stored in liquid nitrogen as previously described (5). The cytokine profile of peripheral blood T cells was determined by flow cytometry and/or ELISpot assay.

### Immunohistochemical Analysis

Punch biopsies from lesional and post-lesional mucosa or skin were obtained from patients 1–4 before treatment and at week 12. A biopsy was further obtained at week 32 from the oral mucosa of patient four. Biopsies were fixed in paraformaldehyde, embedded in paraffin, and further processed for immunohistochemical analysis of the inflammatory infiltrate as recently described (5). Paraffin skin sections were processed by the automated IHC stainer BOND-MAX (Leica, Wetzlar, Germany) and Autostainer Plus automated immunostaining device (Dako, Hamburg, Germany), utilizing the following primary antibodies: mouse anti-human CD3, CD4, CD8 (all Novocastra, Leica, Wetzlar Germany); rabbit anti-human IL-17A, rabbit anti-human FoxP3 (both Novus, Littleton, Co, USA); rabbit anti-human T-bet, rabbit anti-human GATA-3 (both Cell Signaling Technology, Danvers, MA, USA). Secondary antibodies used were: Bond Polymer Refine Detection Kit (Leica, Wetzlar Germany; CD3, CD4, CD8); biotinylated anti-rabbit IgG, biotinylated anti-mouse IgG (Vector Laboratories; Burlingame, CA, USA; IL-17A, FoxP3, T-bet, GATA-3). Antibodies of Vector Laboratories were subsequently detected by peroxidase- or alkaline phosphatase-labeled ABC-systems (Dako). Visualization was carried out using 3,3′-diaminobenzide (DAB) or Liquid Permanent Red staining (both from Dako) as chromogenes. The T cell infiltrate of skin lesions was quantified based on microscopical images (Axiostar, Zeiss, Jena, Germany) in combination with Cell^∧^D (Soft Imaging System, Berlin, Germany) and ImageJ software (Figure S1). At 100x magnification, CD3^+^, CD4^+^, CD8^+^, and IL-17A^+^ T cells were counted (200x magnification for CD3^+^/T-bet^+^ and CD4^+^/GATA-3^+^ T cells). After generating a grid (ImageJ software; area per point: 50.000 pixels^∧^2), all stained T cells were counted in two squares (four squares for CD3^+^/T-bet^+^ and CD4^+^/GATA-3^+^ T cells) adjacent to basal membrane zone (ImageJ, cell counter; Figure S1) and their proportion of all infiltrating cells was determined afterwards. Moreover, with regard to IL-17A^+^ T cells, the T cellular infiltrate adjacent to the dermal-epidermal BMZ was further analyzed on images taken at 200x magnification and after grid formation (ImageJ software; area per point: 50.000 pixels^∧^2) counting four squares in the basal membrane zone and dermal region (ImageJ, cell counter).

### Multiplex Immunohistochemistry Staining

Sections were prepared from a paraffin-embedded biopsy of the oral mucosa of patient 5 which showed the same characteristics with a lichenoid mixed CD4^+^ and CD8^+^ cellular infiltrate as in the other patients. This patient biopsy was chosen because it provided enough material to establish a more refined IF analysis. The Opal multiplex staining utilizes individual TSA conjugated fluorophores to detect multiple targets (Opal 7-Color Manual IHC Kit, Catalog No. NEL811001KT). Firstly, sections were de-paraffinized and fixed in 10% neutral buffered formalin prior to antigen retrieval in heated AR6 buffer for 15 min (EZ Retriever microwave, BioGenex). Each section was put through three sequential rounds of staining, each including a protein block with Opal blocking buffer followed by primary antibody incubation overnight (CD4: 1:100, Abcam; CD8: 1:50, Novus Biologicals; and IL−17: 1:200, R&D) and corresponding secondary horseradish peroxidase-conjugated polymer and Opal fluorophores- CD4 (Opal 620), CD8 (Opal 690), and IL17 (Opal 540). Additionally, DAPI was applied as a nuclear marker. To visualize the location of CD4 and CD8 cells (with IL-17 positive or negative), phenotyping maps were generated based on the aforementioned markers and inform machine-learning algorithms. Furthermore, to create classic pathology views, simulated IHC staining images indicating CD4, CD8, and IL17 expression were generated.

### Blood Cell Isolation

Peripheral blood mononuclear cells of the patients were isolated from citrate-phosphate-dextrose-adenine (CPDA)-treated blood samples and stored in liquid nitrogen as previously described (5).

### Flow Cytometric Analysis of Peripheral Blood Lymphocyte Subsets

After thawing, cells were cultured overnight in RPMI-1640 in RPMI-1640 supplemented with 100 U/ml penicillin, 100 μg/ml streptomycin and 2 mM L-glutamine (all Capricorn, Ebsdorfergrund, Germany) and 10% FCS (Merck Millipore, Berlin, Germany). For detection of leukocyte subsets, cells were stained with the following antibodies: mouse anti-human CD45-AlexaFluor700 (2D1), mouse anti-human CD4-BrilliantViolet510 (RPA-T4; both BioLegend, San Diego, CA, USA), mouse anti-human CD3-PE (UCHT1), mouse anti-human CD8-FITC (SK1), mouse anti-human CD19-PerCP-Cy5.5 (HIB19), mouse anti-human CD14-APC (M5E2; all BD Biosciences, Heidelberg, Germany).

For cytokine detection, cells were stimulated *ex vivo* with 5 ng/mL phorbol myristate acetate (PMA; Promega, Fitchburg, MA, USA) and 500 ng/mL ionomycin (Calbiochem, Billerica, MA, USA) for 5 h at 37°C with addition of GolgiStop (BD Biosciences, Heidelberg, Germany) to block cytokine secretion. Subsequently, cell surface markers were stained using the following antibodies: mouse anti-human CD45-AlexaFluor700 (2D1; BioLegend, San Diego, CA, USA), mouse anti-human CD3-PE-Cy5.5 (SK7; ThermoFisherScientific, Langenselbold, Germany), mouse anti-human CD8-FITC (SK1; BD Biosciences, Heidelberg).

Intracellular cytokines were detected using mouse anti-human IFN-γ-AlexaFluor647 (B27), mouse anti-human IL-21-PE (3A3-N2.1), mouse anti-human IL-17A-AlexaFluor647 (N49-653; all BD Biosciences, Heidelberg, Germany). Cells were acquired on a BD LSRFortessa (BD Biosciences, Heidelberg, Germany) and cell doublets were discriminated by FSC-H/FSC-A plots (Figures S2, S3). Dead cells were excluded from analysis using Zombie NIR staining (BioLegend, San Diego, CA, USA). Data was analyzed by BD FACSDiva Software 8.0.2 (BD Biosciences, Heidelberg, Germany).

### Enzyme-Linked Immunospot (ELISpot) Assay of Peripheral Blood Lymphocytes

ELISpot assays were performed as previously described (5). IFNγ-, IL-5- and IL-17A- positive spots were detected according to the manufacturers' instructions (Human IFNγ-ELISpot, Human IL-5-ELISpot, Becton Dickinson, Franklin Lakes, NJ, USA; Human IL-17A ELISpot Ready-Set-Go, eBioscience, San Diego, CA, USA). PBMC were seeded at 1 × 10^5^ – 5 × 10^5^ cells per well on the ELISpot plates and developed plates were finally analyzed by the ELISpot plate reader A.EL.VIS (A.EL.VIS, Hanover, Germany). For data analysis, the spots of the non-stimulated controls (mean) were subtracted from the spots (mean) of the cultures with antigen (all in duplicate).

## Results

**Figure 1:**
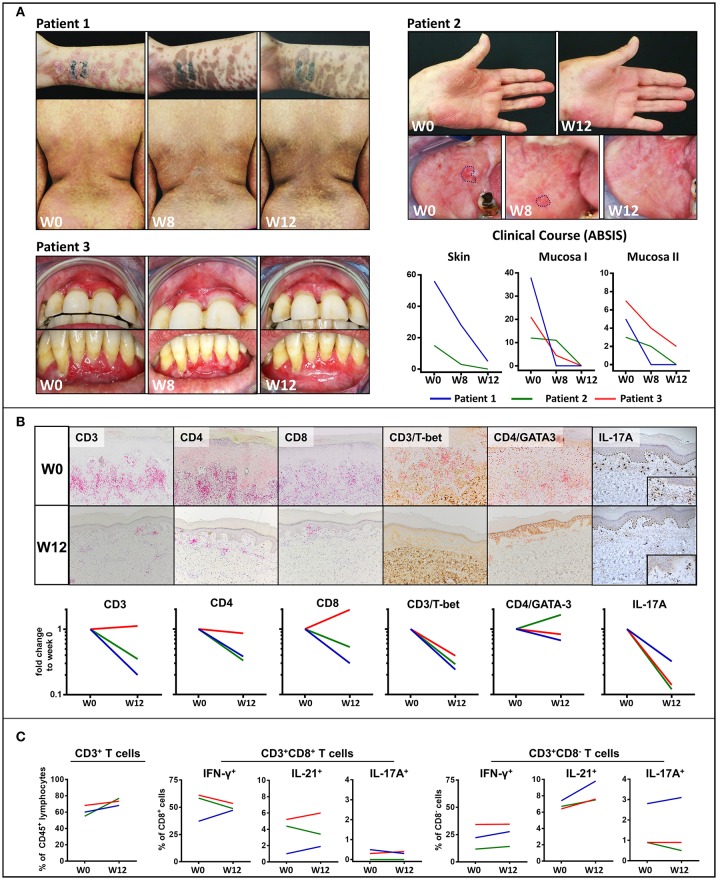
Clinical and immunological response to treatment with the anti-IL-17 monoclonal antibody, secukinumab, in mucocutaneous lichen planus (LP). **(A)** Clinical appearance of three LP patients before (week 0; W0) and on treatment (W8, W12) with secukinumab. Patients 1 and 2 presented with muco-cutanous lesions while patient three had a recalcitrant LP of the oral mucosa unresponsive to systemic immunosuppressive treatment. Blockade of IL-17A led to a shift from erythematous inflammatory to hyperpigmented post-inflammatory skin lesions and an almost complete disappearance of the mucosal lesions by W12. Clinical course was assessed by the Autoimmune Bullous Skin Disorder Intensity Score (ABSIS) showing a marked decrease of cutaneous lesions (ABSIS Skin), mucosal lesions (ABSIS Mucosa I), and substantial improvement of the patients' ability to eat defined foods of increasing consistency without pain and/or bleeding (ABSIS Mucosa II). **(B)** Immunohistochemical analysis of the inflammatory skin infiltrate before (W0) and after 12 weeks (W12) of treatment (representative images of patient 1 are shown). Markers as below: CD3/CD4/CD8 (liquid permanent red), T-bet/GATA3/IL-17 (3,3′ diaminobenzide brown). The dermal, band-like CD4^+^ and CD8^+^ T cellular infiltrate was strongly reduced at W12 of secukinumab treatment compared to W0. Of note, blockade of IL-17A led to a marked decrease of both IL-17A^+^ and CD4^+^/T-bet^+^ (Th1) cells in the LP lesions (see Figure S1 in the supplement for description of image quantification). **(C)** Flow cytometric analysis of peripheral blood CD8^+^ and CD4^+^ (defined as CD8^−^) T cells in the three LP patients before (W0) and at W12 of treatment. In contrast to skin lesions, the number of CD3^+^, IFNγ^+^ (Th1), and IL-17A^+^ peripheral blood T cells remained largely unaffected by blockade of IL-17A. Notably, peripheral blood IL-21^+^ T cells were increased in all the three LP patients (see Figure S2 and Table S1 in the supplement for gating strategy and raw data).

Two patients with extensive cutaneous LP and oral lesions (Patient 1 and 2), respectively, showed rapid clinical improvement on treatment with secukinumab which became apparent by a shift from inflammatory erythematous to post-inflammatory hyperpigmented skin lesions and a regression of oral lesions within 12 weeks (Figure 1A). Moreover, the patient with recalcitrant LP of both the oral mucosa and the tongue (Patient 3) experienced resolution of buccal lesions and lasting improvement of the recalcitrant ulcerative lesions of the tongue upon treatment with secukinumab (Figure 1A). Eventually, lesions of both upper and lower gingiva fully resolved upon long-term follow-up after 48 weeks (Figure S4A). Clinical improvement was reflected by a substantial decrease of ABSIS Skin and ABSIS Mucosa I scores as well as an improvement of functional sequelae of oral involvement (ABSIS Mucosa II). Within 12 weeks, there was a marked reduction of the CD4^+^ and CD8^+^ T cellular skin infiltrate within LP lesions, in particular of Th1 (CD3^+^Tbet^+^) cells (Figure 1B). IL-17A^+^ T cells, which initially lined up along the dermal-epidermal BMZ, were only sparsely found after 12 weeks of secukinumab treatment (Figure 1B). In contrast, secukinumab did not induce major alterations of peripheral blood T cell subsets except for an increase of CD4^+^IL-21^+^ T cells (Figure 1C).

**Figure 2:**
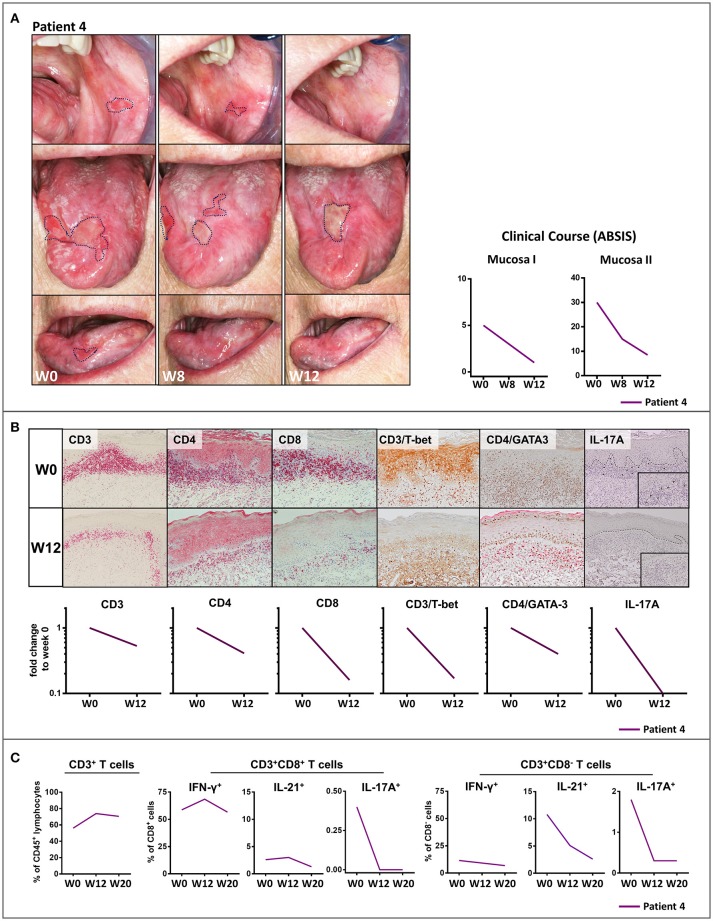
Clinical and immunological response to treatment with the anti- IL-12/IL-23 monoclonal antibody, ustekinumab, patient 4 with recalcitrant oral lichen planus (OLP). **(A)** Clinical appearance before (week 0, W0) and at W8 and W12 of treatment with ustekinumab showing a striking clinical improvement of mucosal lesions by W8 and W12. The clinical course was characterized by a marked reduction of mucosal lesions (ABSIS Mucosa I) and a substantial improvement of the patient's ability to eat defined foods of different consistency without pain and/or bleeding (ABSIS Mucosa II). **(B)** Immunohistochemical analysis of inflammatory mucosal infiltrate before (W0) and at W12 of treatment with ustekinumab. Markers as below: CD3/CD4/CD8 (liquid permanent red), T-bet/GATA3/IL-17 (3,3' diaminobenzide brown). At W12, IL-17A^+^ cells were completely absent and the CD4^+^T-bet^+^ (Th1) cell infiltrate was strongly diminished (see Figure S1 for description of image quantification). **(C)** Flow cytometric analysis of peripheral blood CD8^+^ and CD4^+^ (defined as CD8^−^) T cells before (W0) and at W12 and W20 of ustekinumab treatment. While peripheral blood CD3^+^ T cells were not impaired, blockade of IL-12/IL-23 led to a strong decrease of peripheral blood IL-17A^+^ and IL-21^+^ T cells (see Figure S3 and Table S1 in the supplement for gating strategy and raw data).

**Figure 3:**
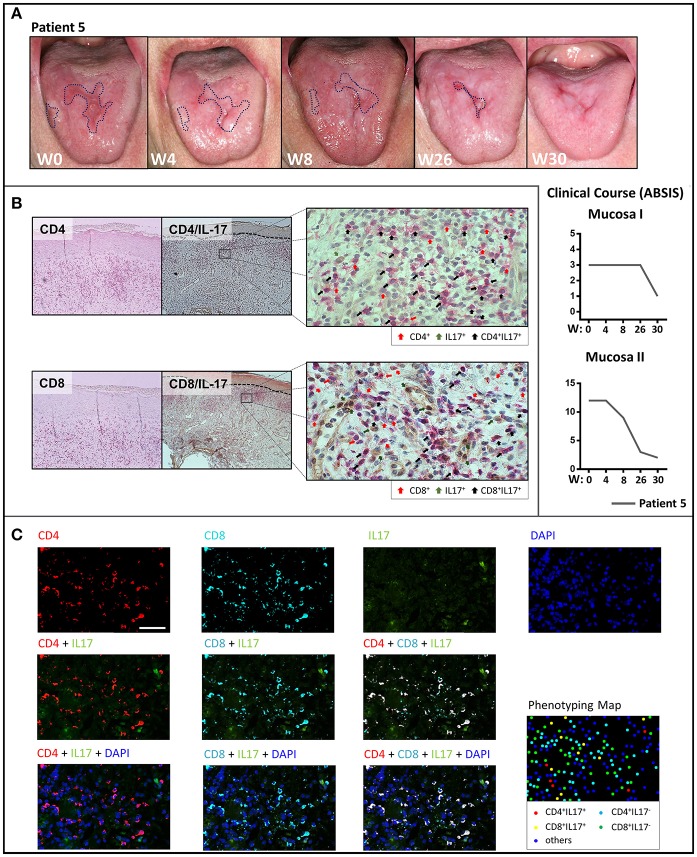
Clinical response of oral lichen planus to treatment with guselkumab and phenotypical analysis of the T cell infiltrate by immunohistochemistry and multiplex immunohistochemistry. **(A)** Clinical appearance of the oral mucosa with marked ulcerative lesions (dotted blue line) of patient 5 on treatment with guselkumab before treatment at week 0 (W0) and at W4, W8, W26, and W30 with the clinical course assessed by ABSIS score. There is a complete resolution of the ulcerative lesions of the tongue by W30. **(B)** Immunohistochemical staining for CD4, CD8, and IL-17 before treatment with guselkumab. Single staining with CD4^+^, CD8^+^ (red), and IL-17 (green) as well as double positive-staining of CD4^+^ or CD8^+^ T cells with IL-17 (black) are highlighted by arrows. **(C)** Multiplexed immunohistochemistry images of skin lesions showing each of the individual markers in the composite image after spectral unmixing. Markers are as below: CD4 (Opal 620, pseudocoloured red), CD8 (Opal 690, pseudocoloured cyan), IL−17 (Opal 540, pseudocoloured green), and DAPI as a nuclear marker (blue) (upper row), Multiplexed immunohistochemistry images of CD4 or/and CD8 staining merged with IL-17 (middle row). Multiplexed immunohistochemistry images of CD4 or/and CD8 along with IL-17 merged with DAPI (lower row). The cell phenotyping map identifies CD4^+^IL17^+^ cells (red dots), CD8^+^IL17^+^ cells (yellow dots), CD4^+^IL17^−^ cells (cyan dots), CD8^+^IL17^−^ cells (green dots), and other cells (blue dots).

Treatment with the anti-p40 monoclonal antibody, ustekinumab of a patient with recalcitrant ulcerative oral LP (Patient 4) led to dramatic clinical improvement within 12 weeks (Figure 2A). The buccal lesions fully resolved and the number and size of lesions of the tongue were markedly reduced which was also reflected by improved ABSIS Mucosa I and II scores. The inflammatory cutaneous T cell infiltrate which was mainly composed of Th1 (CD3^+^T-bet^+^) and IL-17A^+^ T cells markedly (Th1) or completely (IL-17A^+^) resolved within 12 weeks of ustekinumab treatment (Figure 2B). On long term follow up, the ulcerative lesions of the tongue and mucosal lesions of the oral region completely fully resolved by week 48 (Figure S4B). In contrast to secukinumab, treatment with ustekinumab resulted in a strong reduction of peripheral blood IL-17A^+^ as well as IL-21^+^ T cells (Figure 2C). Overall, secukinumab and ustekinumab treatment had no major impact on other peripheral blood leukocyte subsets (Figure S5). Noteworthy, secukinumab did not inhibit the number of IL-17-producing T cells in the peripheral blood of patients while ustekinumab induced a continuous decline of IL-17-secreting T cells during treatment (Figure S6). Moreover, treatment of a patient with recalcitrant erosive and ulcerative LP of the tongue with the anti-IL-23 monoclonal antibody guselkumab (Patient 5) led to a constant amelioration of oral erosions which fully resolved by week 30 (Figure 3A).

To further determine the cellular source of IL-17A in the lesional skin of LP, we performed multiplex immunohistochemistry (Figure 3C) in addition to regular immunohistochemical analysis in this patient (Figure 3B). We here identified several positive Il-17^+^ T cells within both, the CD4^+^ and CD8^+^ T cell infiltrate, in support of a mixed Th17/Tc17 infiltrate in LP lesions (Figure 3C).

## Discussion

We here show for the first time that therapeutic targeting of Th17/Tc17 cells either by blocking IL-17A with the monoclonal antibody, secukinumab, or by inhibiting the Th17/Tc17 axis with ustekinumab (anti-IL-12/IL-23) or with guselkumab (anti-IL-23) leads to a marked and prolonged improvement of mucosal and cutaneous lesions in LP. This finding is a major therapeutic advance in LP which often shows a chronic, refractory course and has a major impact on the patients' quality of life (1). Blockade of IL-17 and inhibition of Th17/Tc17 cell expansion was equally effective in reducing mucosal and cutaneous LP lesions. Thus, Th17/Tc17 cells and T cell-derived IL-17A are presumably important for disease induction and probably also for the perpetuation of the inflammatory response in LP. The concept that Th17/Tc17 cells are the major source of IL-17 in LP is supported by the finding that (1) CD4^+^/IL-17^+^ and CD8^+^/IL17^+^ cells line up along the dermal-epidermal BMZ in close proximity to apoptotic epidermal keratinocytes (5), a hallmark of LP, (2) neutrophils are virtually absent in LP, and (3) γδ-T cells have been only occasionally and inconsistently described in LP skin lesions and lesion-derived T cell lines (14, 15). However, other cell populations that can produce IL-17 (e.g., NK cells, NKT cells, and γδ T cells), which were not analyzed in this study, may also be a source of IL-17. However, using multiplex immunohistochemistry we mainly observed CD4^+^ or CD8^+^ IL-17A-producing T cells pointing toward an implication of Th17 and Tc17 in LP pathogenesis (Figure 3C). Thus, based on these findings, we here specifically addressed the potential role of IL17^+^ T cells in LP pathogenesis and the impact of therapeutic targeting of Th17/Tc17 cells.

Secukinumab, ustekinumab, and guselkumab were chosen for compassionate use in the studied patients with extensive or recalcitrant LP because they are licensed for the treatment of the Th17-associated inflammatory disorders, psoriasis vulgaris, psoriatic arthritis and Crohn's disease (16, 17). Th17 blockade also holds promise in the treatment of multiple sclerosis and rheumatoid arthritis (18), which are also strongly linked to a Th17-dependent pathogenesis.

A pathogenetic role of IL17 in LP had been previously suggested by several independent studies based on the detection of elevated serum concentrations of IL-17 and increased numbers of peripheral and lesional Th17 cells in patients with mucocutaneous and oral LP (7–9). Furthermore, the Th17-derived cytokines, IL-21, IL-22, and IL-23 were found to be upregulated in LP lesions (6, 7) Furthermore, also serum levels of IL17A seem to be upregulated in patients with LP (19). Recently, we showed that patients with LP have autoreactive peripheral blood Th17 cell responses against bullous pemphigoid (BP) 180, the autoantigen of BP (5). BP is considered as a mainly Th2-driven, IgG-mediated autoimmune disorder against the hemidesmosomal component, BP180, and presents with a Th2-dominated inflammatory skin infiltrate. In contrast, patients with LP showed a pronounced Th1/Th17-dominated infiltrate in LP lesions and peripheral blood autoreactive Th1/Th17 cells (5).

Th17 cells are characterized by the expression of the transcription factor, RORγt, and secretion of the signature cytokine IL-17A and other Th17-derived cytokines such as IL-17F, IL-21, and IL-22 (20). Based on the cellular and immunological context, Th17 cells display a great degree of plasticity (20, 21). Under non-pathogenic conditions, Th17 contribute to proper epithelial barrier function and the defense of fungal (Candida) or bacterial (staphylococci) pathogens (20, 22). Under pro-inflammatory conditions, Th17 cells can trans-differentiate into anti-inflammatory T regulatory type 1 (Tr1) cells in the intestine (23) whereas in experimental models of inflammatory bowel disease and multiple sclerosis, Th17 cells have been shown to also promote pro-inflammatory pathogenic immune responses by gaining IFN-γ and/or GM-CSF production (20, 24). Moreover, Th17 cell-derived IL-17 induces, in concert with IL-6 and TGF-β, a positive feedback loop that further drives Th17 cell expansion (25). Of note, pathogenic Th17 and presumably also Tc17 cells can rapidly transform into IFN-γ-secreting non-classical Th1 cells (26–28). Thus, the Th1-dominated T cellular infiltrate in LP may be at least partly Th17/Tc17 cell-derived which is supported by the marked decrease not only of Th17 but also Th1 cells in LP lesions after IL-17A blockade by secukinumab (Figure 1B). Blockade of IL-17 may prevent the transformation of pathogenic Th17 cell-derived non-classical Th1 cells. Accordingly, direct blockade of the Th17/Tc17 cell-axis with ustekinumab (anti-IL-12/IL-23) in a patient with refractory oral LP was not only linked to the disappearance of Th17 cells in LP lesions but also to a marked reduction of the lesional Th1 infiltrate (Figure 2B). IL-23 has been described as one of the key drivers of pro-inflammatory pathogenic Th17 cells mainly via induction of the B lymphocyte-induced maturation protein 1 (BLIMP1) (29, 30). Treatment with ustekinumab and guselkumab might therefore be more effective than secukinumab in suppressing Th17/Tc17 and, consecutively, Th1 cells in LP. In fact, in experimental Th17 cell-driven colitis blockade of IL-23 was shown to be superior in down-regulating inflammation than inhibition of soluble IL-17 (31).

Our observations strongly suggest that IL-17 is a critical cytokine in the immune pathogenesis of LP and thus represents a major therapeutic target. Under inflammatory conditions, IL-17 induces chemokine release from various cell types of the skin, including endothelial cells, macrophages, and keratinocytes leading to tissue remodeling and the recruitment of pro-inflammatory effector cells in the skin (32, 33). In LP, IL-17 release in the skin could facilitate initiation of a pro-inflammatory cascade leading to recruitment of T cells which are critical in the pathogenesis of LP. The fact that IL-17 blockade with secukinumab did not show systemic suppressive effect on peripheral Th17/Tc17 cells (Figure 1C) while suppressing the T cellular skin infiltrate further supports a pathogenic role of IL-17 in LP lesions. Instead, there was an increase of IL-17-secreting peripheral blood T cells in two patients (Figure S6) and a slight increase of peripheral blood CD4^+^ T cells producing IL-21 (Figure 1C), an essential inducer of IL-17 secretion which is presumably a compensatory mechanism for loss of secreted IL-17. In contrast, ustekinumab treatment led to a marked reduction not only of peripheral blood Th17/Tc17 cells but also of Th1 cells (Figure 2C).

In summary, we demonstrate for the first time that therapeutic targeting of Th17/Tc17 cells in LP either by blocking IL17A with the anti-IL-17 monoclonal antibody, secukinumab, or by inhibiting the Th17/Tc17 axis with ustekinumab (anti-IL-12/IL-23) or guselkumab (anti-IL-23), leads to a dramatic and prolonged improvement of mucosal and cutaneous lesions in five patients with recalcitrant LP. Our observations strongly suggest a crucial role Th17/Tc17 cells in LP pathogenesis and provides novel therapeutic targets for therapeutic control of this chronic refractory skin disorder.

## Data Availability

The raw data supporting the conclusions of this manuscript will be made available by the authors, without undue reservation, to any qualified researcher.

## Ethics Statement

Compassionate use of specific drugs are warranted in Germany for treatment of up to three patients who have not shown sufficient responses of their disease to approved drugs or with contraindications to treatment with a approved drug. This was the case in the described patients with muco-cutaneous lichen planus. Prior to treatment of the patients with either secukinumab, ustekinumab, or guselkumab. The head of the local ethics committee at Marburg University confirmed that compassionate treatment of these patients with secukinumab, ustekinumab, or guselkumab did not require formal approval by the ethics committee as long as treatment was restricted to a total of three patients for each drug. The patients were explained in detail why we considered compassionate use of these drugs in their situation and they fully approved of this. This entire process was thoroughly documented in the patients' charts.

## Author Contributions

MH, FS, RP, and TS: study concept and design. XZ and RS: multiplexed immunohistochemistry staining and analysis. RP, FS, and MH: drafting of the manuscript. RP: statistical analysis. AS, SM, JP, VE, and CM: administrative, technical, or material support. MH: study supervision. All authors: acquisition, analysis, and interpretation of data and critical revision of the manuscript for important intellectual content.

### Conflict of Interest Statement

MH has been an advisor and speaker for Novartis and a speaker for Janssen Cilag. The remaining authors declare that the research was conducted in the absence of any commercial or financial relationships that could be construed as a potential conflict of interest.
