# Supplementary material for: Therapeutic Targeting of Th17/Tc17 Cells Leads to Clinical Improvement of Lichen Planus
Source: Front Immunol. 2019 Jul 31;10:1808. doi: 10.3389/fimmu.2019.01808 (PMC6685396; doi:10.3389/fimmu.2019.01808)
Supplement: Supplementary file 1 [file Data_Sheet_1.docx]

Supplementary Material

**Therapeutic targeting of Th17/Tc17 cells leads to clinical improvement of lichen planus**

**Farzan Solimani, Robert Pollmann, Thomas Schmidt, Ansgar Schmidt, Xiang Zheng, Rajkumar Savai, Stefan Mühlenbein, Julia Pickert, Verena Eubel, Christian Möbs, Rüdiger Eming, Michael Hertl***

*** Correspondence:** Michael Hertl: [hertl@med.uni-marburg.de](mailto:hertl@med.uni-marburg.de)

**Supplementary Figures**

**
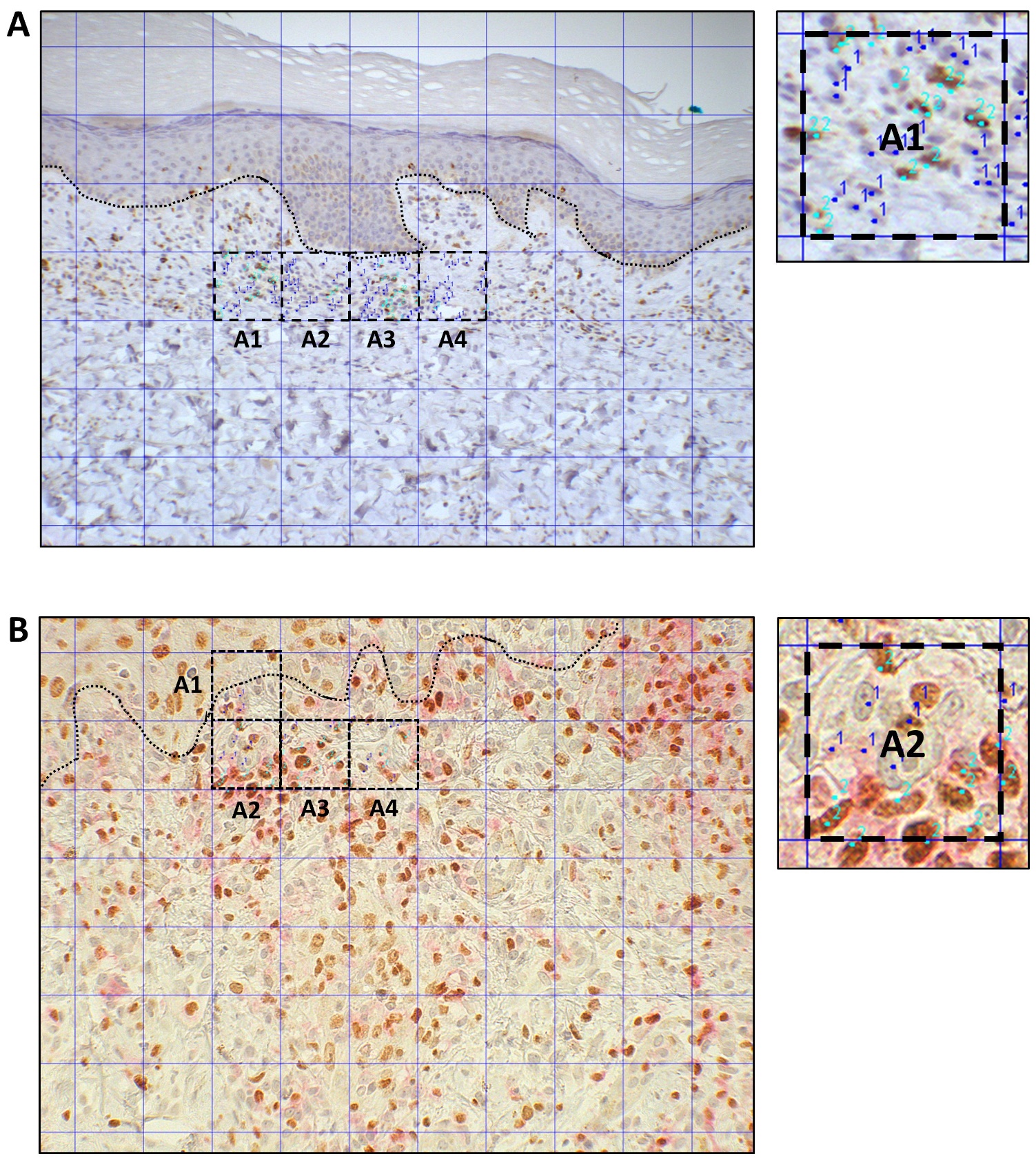
**

**Supplementary Figure 1**

**Analysis of T-lymphocyte subsets in skin lesions of patients with LP treated with secukinumab or ustekinumab.**

Representative evaluation of IL-17A^+^ (**A**), and of CD4^+^GATA3^+^ (**B**) dermal infiltrating cells are shown. Enumeration of T cells was based on use of a microscope (Axiostar, Zeiss) in combination with Cell^D (Soft Imaging System) and ImageJ software. For counting of specifically stained T cells, images of skin lesions at x200 magnification were taken. After generating a grid (ImageJ software; area per point, 50,000 square pixels), all infiltrating and specifically stained cells were counted in 4 defined squares (A1/A2/A3/A4) adjacent to the dermal-epidermal BMZ (ImageJ software, cell counter), and the proportion of all infiltrating cells to specifically stained T cells was determined.

**Supplementary Figure**
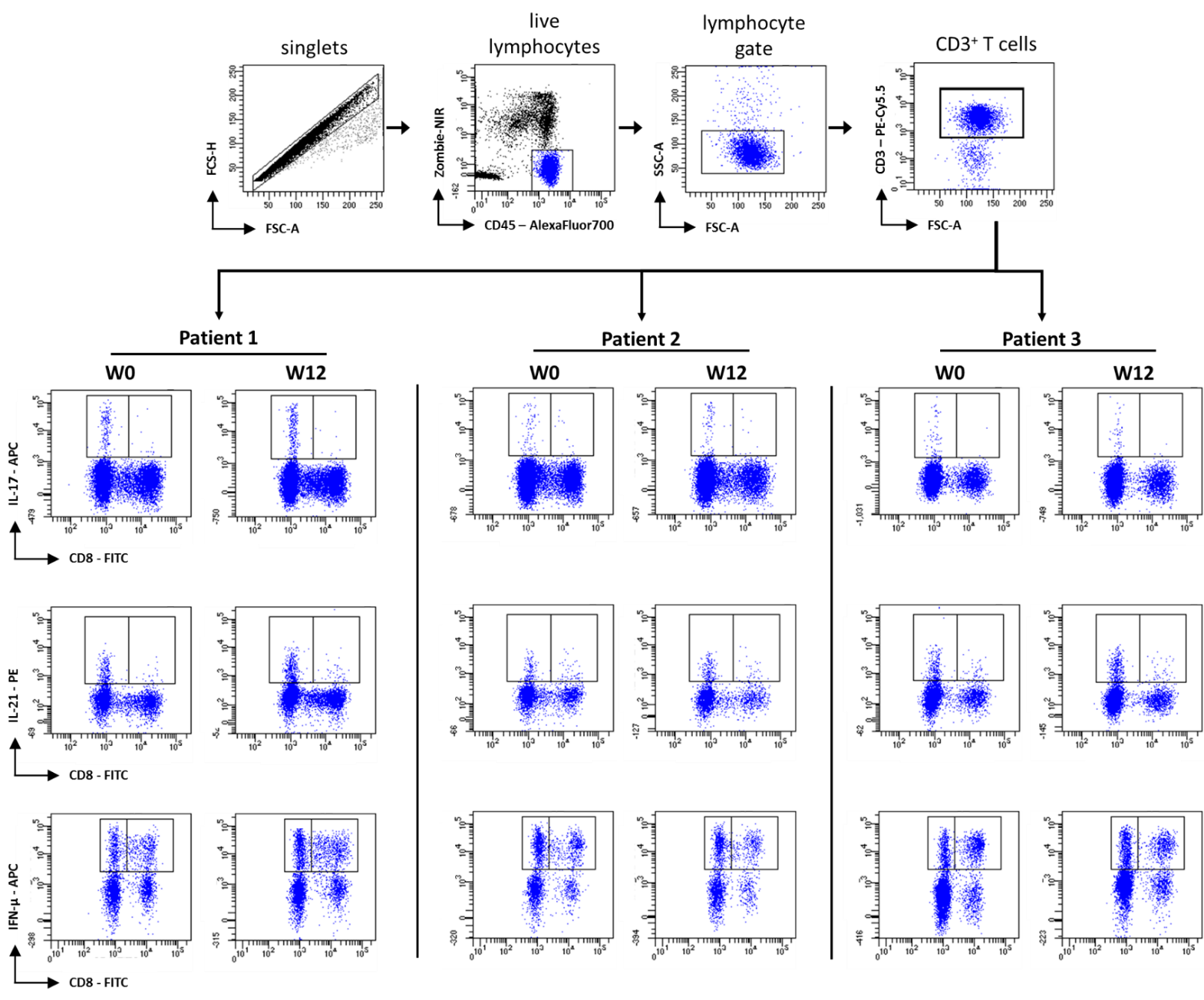
**2**

**Flow cytometric analysis of peripheral blood lymphocyte subsets in patients with lichen planus treated with ustekinumab.**

Individual dotplots for lichen planus patients 1,2, and 3 treated with secukinumab are shown. Gating strategy: cell doublets were discriminated by FSC‑H/FSC‑A plot (singlets gate). Live CD45^+^ lymphocytes were gated based on Zombie NIR staining as well as cell size and granularity (FSC‑A/SSC-A plots). Cytokine production of CD3^+^ T cells was analyzed within CD8^+^ and CD8^-^ T cells for IL-17, IL-21 and INF-µ.


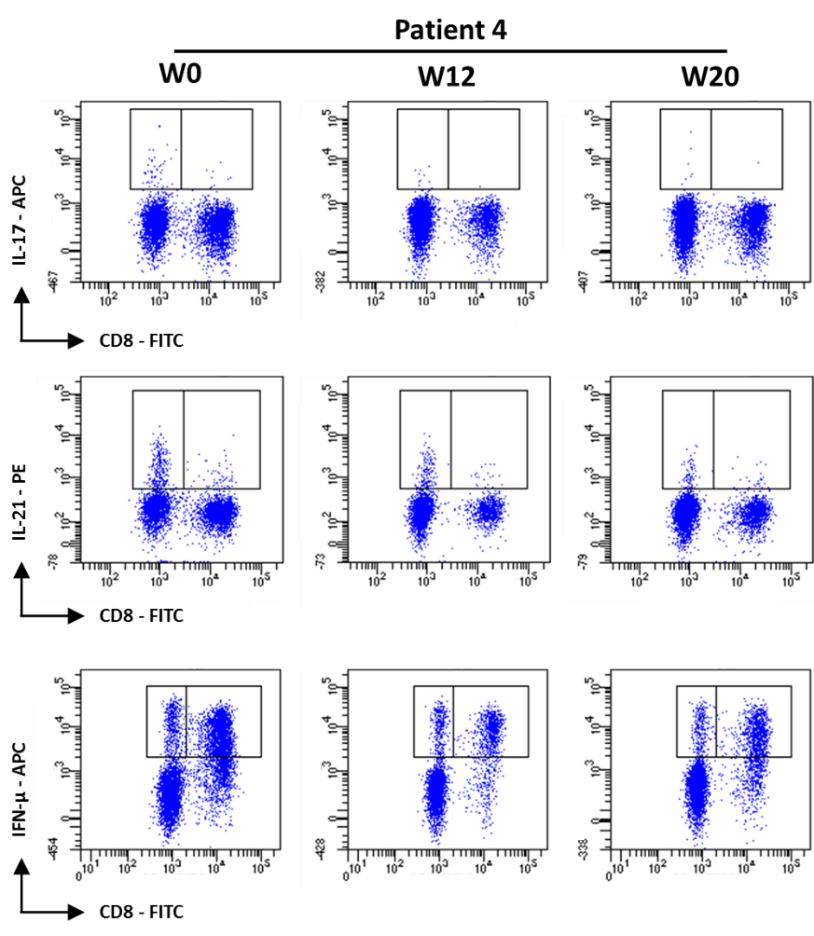


**Supplementary Figure 3**

**Flow cytometric analysis of peripheral blood lymphocyte subsets in patients with lichen planus treated with ustekinumab.**

Individual dotplots for lichen planus patient 4 treated with ustekinumab are shown. Cells were gated according to gating strategy presented in Figure S2. Cytokine production of peripheral blood CD3^+^ T cells was analyzed within CD8^+^ and CD8^-^ T cells for IL-17, IL-21 and INF-γ.

**
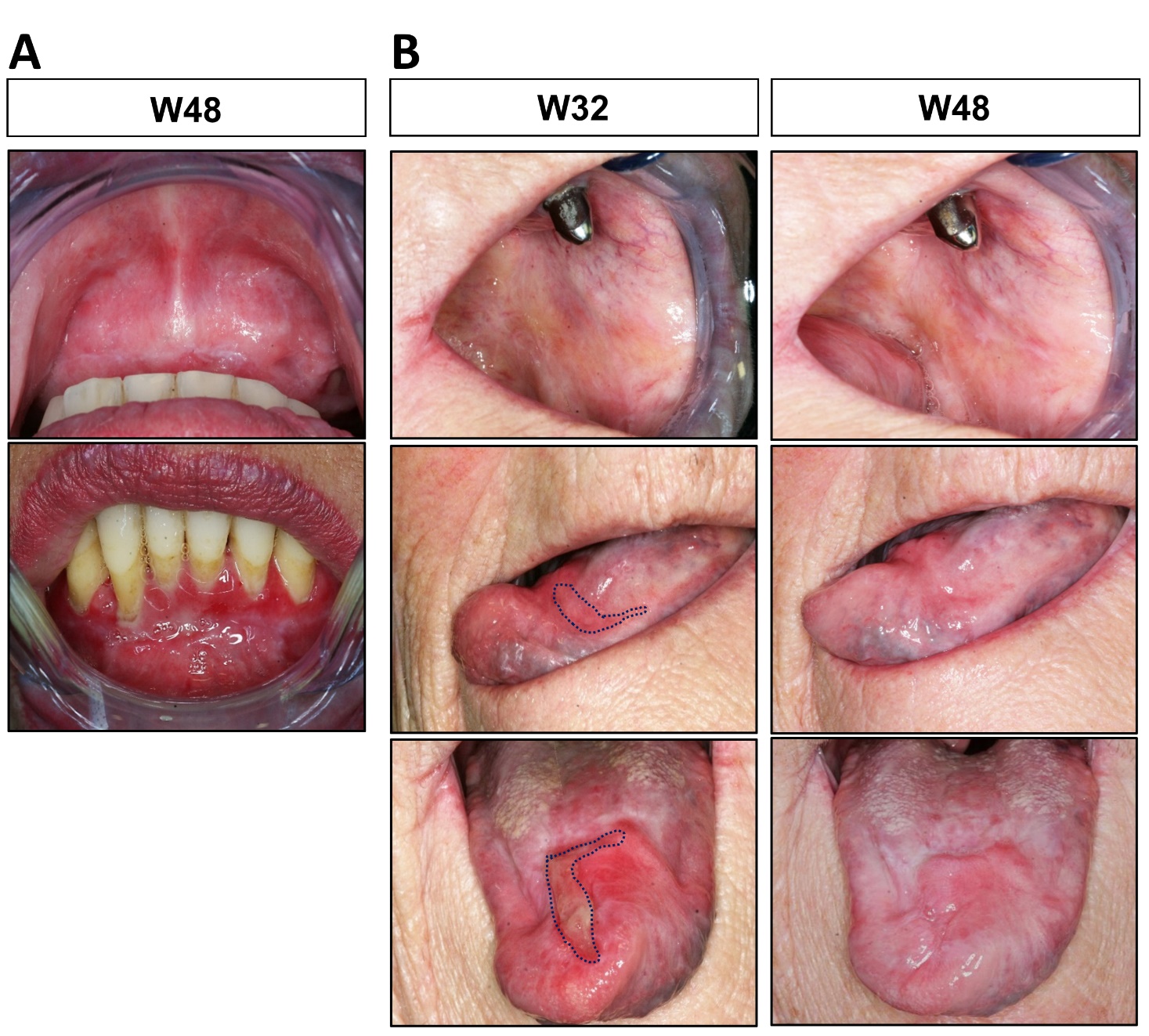
**

**Supplementary Figure 4.**

**Long-term clinical response of patients with lichen planus to treatment with secukinumab and ustekinumab, respectively.**

**(A)** clinical appearance of the oral mucosa of patient 3 at 48 weeks (W48) of treatment with secukinumab. As by week 12, oral erosions had almost completely resolved leaving superficial residual erosions at the lower gingival mucosa. **(B)** clinical appearance and histopathology of the oral mucosa of patient 4 at 32 weeks (W32) and W48 of treatment with ustekinumab. Marked regression of ulcerative lesions of the tongue (dotted blue line) and buccal lesions by W32 and complete resolution of the lesions by W48.

**
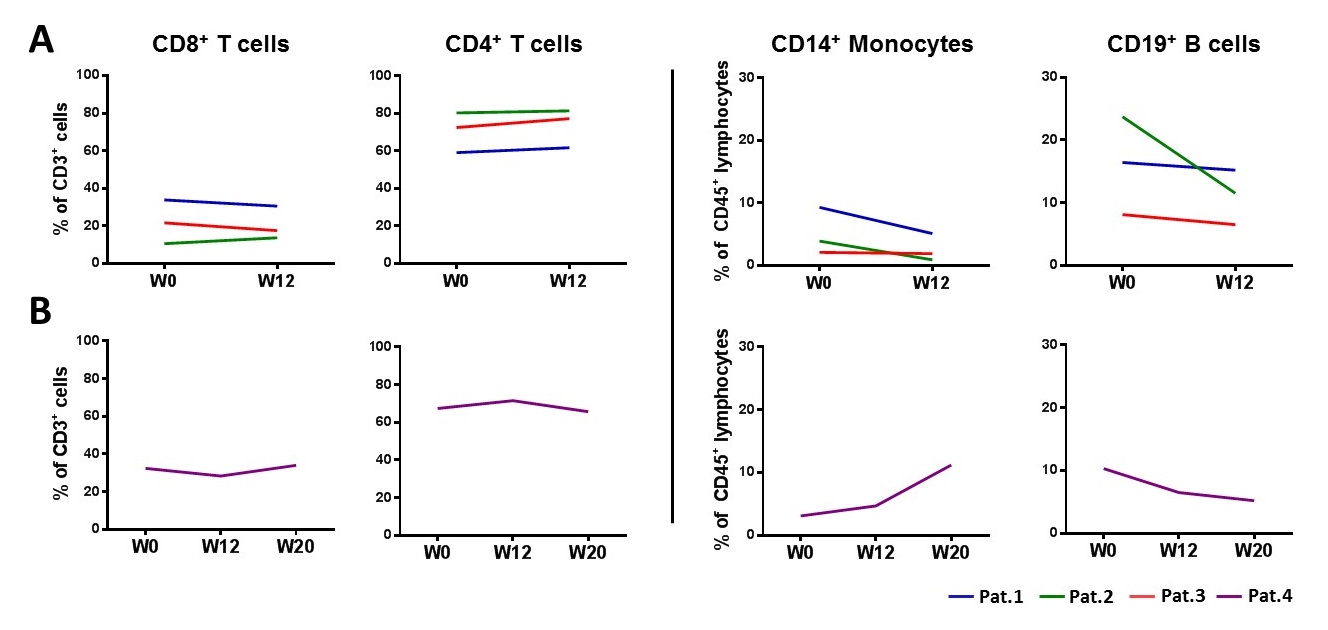
**

**Supplementary Figure 5.**

**Distribution of peripheral blood leukocyte subsets in patients with lichen planus on secukinumab and ustekinumab treatment, respectively.**

Patients 1-3 were treated with the anti-IL-17 monoclonal antibody, secukinumab, and patient 4 was treated with the monoclonal anti-IL-12/IL-23 antibody, ustekinumab. (**A)** percentages of CD8^+^ and CD4^+^ T cells within the peripheral blood CD3^+^ T cell population before (week 0, W0) and at W12 and W20 of treatment. (**B)** percentages of CD14^+^ monocytes and CD19^+^ B cells within the CD45^+^ lymphocyte population before W0 and at W12 and W20 of treatment.

**
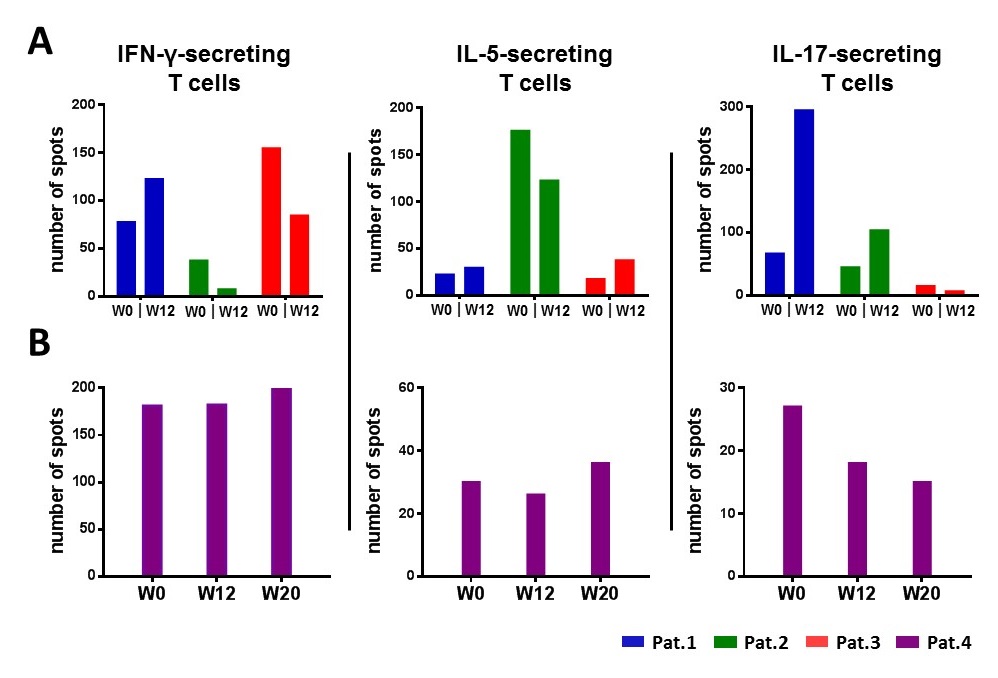
**

**Supplementary Figure 6.**

**Cytokine secretion of peripheral blood T cells in patients with lichen planus (LP) on secukinumab and ustekinumab treatment, respectively.**

Patients 1-3 were treated with the anti-IL-17 monoclonal antibody, secukinumab, and patient 4 was treated with the monoclonal anti-IL-12/IL-23 antibody, ustekinumab. **(A)** ELISpot analysis of IFN-γ-, IL-17A-, and IL-5- secreting T cells upon PHA mitogenesis in LP patients 1-3 before (week 0; W0) and at W12 of secukinumab treatment. IL-17A-secreting T cells increase in patient 1 and 2. (**B)** IFN-γ-, IL-17A-, and IL-5- producing T cells in LP patient 4 before W0 and at W12 and W20 of ustekinumab treatment.

**Supplementary Tables**

|  | **IL-17** | | | | **IL-21** | | | | **IFN-µ** | | | |
| --- | --- | --- | --- | --- | --- | --- | --- | --- | --- | --- | --- | --- |
|  | *W0* | | *W12* | | *W0* | | *W12* | | *W0* | | *W12* | |
|  | CD8^+^ | CD8^-^ | CD8^+^ | CD8^-^ | CD8^+^ | CD8^-^ | CD8^+^ | CD8^-^ | CD8^+^ | CD8^-^ | CD8^+^ | CD8^-^ |
| **P1** | 0.5 | 2.8 | 0.3 | 3.1 | 1.0 | 7.4 | 1.9 | 9.8 | 37.2 | 22.1 | 47.3 | 27.8 |
| **P2** | 0.3 | 0.9 | 0.4 | 0.9 | 5.2 | 6.4 | 6.0 | 7.6 | 61.1 | 34.3 | 53.5 | 34.6 |
| **P3** | 0.0 | 0.9 | 0.0 | 0.5 | 4.4 | 6.7 | 3.4 | 7.5 | 58.4 | 11.7 | 48.8 | 14.2 |

|  | **IL-17**  **IL-21** | | | | | | | | | | **IL-21** | | | | | |
| --- | --- | --- | --- | --- | --- | --- | --- | --- | --- | --- | --- | --- | --- | --- | --- | --- |
|  | *W0* | | | *W12* | | | | *W20* | | | *W0* | | *W12* | | *W20* | |
|  | CD8^+^ | | CD8^-^ | CD8^+^ | | | CD8^-^ | CD8^+^ | | CD8^-^ | CD8^+^ | CD8^-^ | CD8^+^ | CD8^-^ | CD8^+^ | CD8^-^ |
| **P4** | 0.4 | | 1.8 | 0.01 | | | 0.3 | 0.01 | | 0.3 | 2.6 | 10.8 | 3.0 | 5.1 | 1.3 | 2.6 |
|  | **IFN-µ** | | | | | | | | | |  |  |  |  |  |  |
|  | *W0* | | | | *W12* | | | *W20* | | |  |  |  |  |  |  |
|  | CD8^+^ | CD8^-^ | | | CD8^+^ | CD8^-^ | | CD8^+^ | CD8^-^ | |  |  |  |  |  |  |
| **P4** | 58.7 | 11.5 | | | 68.5 | 9.2 | | 56.6 | 6.8 | |  |  |  |  |  |  |

**Supplementary Table 1**

**Flow cytometric analysis of peripheral blood lymphocyte subsets in patients with lichen planus treated with secukinumab and ustekinumab**

Cytokine production by peripheral T cells was determined by flow cytometry (see Figure S2 for gating strategy) in patients treated with secukinumab (P1,P2,P3) and ustekinumab (P4) before treatment week 0 (W0) and after treatment at week 12 (W12) or W20, respectively, as presented in Figure 1C and Figure 2C. Shown are the percentage of IL-17^+^, IL-21^+^, and IFN-µ^+^ T cells within the CD3^+^CD8^-^ and CD3^+^CD8^+^ cell compartment. Dotplots for each individual patients are shown in Figure S2 and S3.
